# Psychological aspects of infertility

**DOI:** 10.1515/medgen-2024-2029

**Published:** 2024-09-06

**Authors:** Tewes Wischmann

**Affiliations:** Heidelberg University Hospital Institute of Medical Psychology Bergheimer Str. 20 69115 Heidelberg Germany

**Keywords:** infertility, psychosocial aspects, assisted reproduction, third-party reproduction, infertility counselling, Infertilität, psychosoziale Aspekte, assistierte Reproduktion, Familienbildung mithilfe Dritter, Kinderwunschberatung

## Abstract

The unfulfilled desire for children is a significant problem worldwide. The psychological effects of this development are usually underestimated, while the myth of “psychogenic infertility” stubbornly persists. This article first provides an overview of the basic facts on the subject before highlighting the psychological effects of both the diagnosis of infertility and the therapeutic options. Psychological aspects of “third-party” reproduction and further developments after childbirth or without a child are discussed, followed by a brief outline of the general and specific subject matter addressed in infertility counselling. The article concludes with reflections on the possible psychological consequences of further medical developments in this area.

## Zusammenfassung

Unerfüllter Kinderwunsch ist weltweit ein bedeutsames Problem. Die psychologischen Auswirkungen dieser Entwicklung werden meist unterschätzt, während der Mythos der „psychogenen Infertilität“ hartnäckig persistiert. In diesem Beitrag wird zunächst eine Übersicht über grundlegende Fakten zur Thematik gegeben, bevor die psychologischen Auswirkungen sowohl der Diagnosestellung Infertilität als auch der therapeutischen Optionen aufgezeigt werden. Psychologische Aspekte der Familienbildung mit Hilfe Dritter und der weiteren Entwicklung nach Kindsgeburt bzw. ohne Kind werden skizziert, wie auch ein kurzer Einblick in allgemeine und spezifische Beratungsinhalte bei unerfülltem Kinderwunsch gegeben. Der Beitrag schließt mit Überlegungen zu möglichen psychologischen Konsequenzen der weiteren medizinischen Entwicklungen in diesem Bereich.

## 1 Basic facts and figures on infertility and assisted reproduction

### 1.1 Prevalence of involuntary childlessness

According to the WHO’s definition, it is appropriate to speak of involuntary childlessness when despite regular unprotected sexual intercourse a couple has been waiting one year or more for pregnancy [54]. However, this definition covers only the (unfulfilled) desire for a child in heterosexual couples, i. e. the “biomedical barriers” [23]. It leaves out of account both “social barriers” (e. g. lesbian couples, queer couples and partnerless individuals desiring a child) and “situational barriers” (ongoing contraception despite the desire for a child occasioned by external factors perceived as an obstacle, e. g. straitened financial circumstances). Nor is it clear how couples are to be assessed if one of the partners wants a child and the other does not (or not yet). These examples indicate how difficult it is to supply precise figures on the prevalence of involuntary infertility.

According to ESHRE, some 25 million people in the EU are affected by infertility. In the course of time, this figure has risen from about 9 % [3] to approx. 15 % of the population [12]; the WHO assesses the lifetime prevalence of infertility in Europe to be 16.5 % [8]. In Germany, an increase in infertility can also be assumed. In a survey conducted in 2013, one out of four childless couples indicated that this situation was involuntary. In 2019 the proportion had risen to one couple in three [38] [39]. The main reason for this is the increase in the average age of women giving birth for the first time. In 2022 this was 30.4 years (29.2 in 2012) [9]. The woman’s biological age is still held to be the most important predictor for human fertility [18].

### 1.2 Potentialities and risks of ART

In 2019, some 1.08 million ART treatment cycles were carried out in Europe, of which approx. 160,000 were IVF cycles, approx. 428,000 ICSI cycles (“fresh” transfers in both cases), 335,000 cycles with frozen oocytes (“cryo cycles”) and 82,000 cycles with oocyte donation [26]. For that same year all over Europe, approx. 147,000 inseminations (IUIs) with partner semen and some 51,000 inseminations with donor semen were additionally reported. However, approx. one out of seven of the reproductive medicine centres participating in the register reported no data for 2019. Accordingly, it is difficult to assess the success rates actually achieved, i. e. the birth rate per treatment cycle carried out (subsequent to punctation resp. thawing of oocytes). The average birth rates reported in the German register for 2019 are 14.9 % per IVF/ICSI cycle (“fresh” transfers taken together) and 13.2 % per cycle initiated [26, Tab. 3].

From the birth rates per ART cycle we can calculate the cumulative birth rate. After three attempts with IVF/ICSI, some 50 % of the couples on average (a 20 % per cycle birth rate assumed) are still unsuccessful in terms of the target outcome (childbirth), and after five attempts the figure is still approx. one-third [27] (under optimal conditions, these figures can be significantly more positive: a 30-year-old patient with secondary infertility and a desire for a child for two years can expect a cumulative birth rate of around 75 % after three ICSI cycles [24]). For cryo treatments and IUIs, the cumulative rates are somewhat lower, for oocyte donation significantly higher. Accordingly, a large majority of couples undergoing treatment with ART need to face up to the fact that the undertaking is likely to remain unsuccessful [15] and would be well advised to devise a “plan B” in good time [52].

One of the most important risks involved in ART treatment is the likelihood of multiple births. In Europe in 2019, 11.9 % of all births following “fresh” IVF/ICSI cycles were twin births and 0.3 % triplet births [26]. That means that in comparison with birth after spontaneous conception, the rate of twins is approx. 10 times higher and the rate of triplets 22 times higher. The count for Europe as a whole lay between 0.0 % multiple births in Iceland and 30.7 % in Albania. In Germany in 2019, the rate of twin births was 18.1 % and that of triplet births 0.4 %. Despite recent major improvements in obstetric intervention resources, the health risks for multi-birth progeny should not be underestimated. Compared with singletons, the risk of death by age 1 year is 7 times higher for twins and 20 times higher for triplets [42]. Over half of the twins and just over 90 % of the triplets born after ART treatment in 2019 were premature [26, Tab. S15].

## 2 Psychological impact of infertility and diagnosis

The confrontation with infertility is bound up with specific psychological challenges (for an overview see the guideline [42]). The diagnosis normally finds couples unprepared and is in addition an “invisible” loss. Long-term plans for their later lives together have to be abandoned and couples have no well-tried mourning rituals at their disposal. The experience of “physical failure” (= not getting pregnant) is normally compounded with the experience of “coping failure” as the emotions involved are frequently experienced as overcharged and uncontrollable. Given the stigmatization of the topic, infertile persons have no lobby, and women and men cannot fall back on role models in attempting to come to terms with the crisis. Frequently, they have no access to specialized, low-threshold counselling by qualified professionals. In counselling for such couples, these specific aspects need to be taken into account [47]. In terms of the emotional effects involved, many of those affected by infertility feel them to be as severe as those caused by serious illness or the loss of a close relative [10]. Accordingly, playing down the significance of involuntary childlessness is likely to be counterproductive. Counsellors should bear this in mind and proceed accordingly.

At a superficial level, men and women appear to vent their distress in different ways. Whereas women give emotional expression to their grief and readily talk about it, men tend to avoid overt expressions of emotion and assume the role of the “stoical partner” [50]. Probably all individuals, male or female, have at their disposal (albeit to different degrees) both loss-oriented and recovery-oriented coping styles not necessarily bound up with traditional gender roles [51].

Some coping strategies are more effective in dealing with the experience of infertility-specific stress than others. Active-avoidant coping seems to be the least helpful strategy in connection with emotional adaptation, with meaning-based coping apparently the most favourable alternative [13]. This also applies to the way couples square up to (recurrent) miscarriage [36]. Psychological counselling could help to strengthen the use of positive coping strategies (such as meaning-based coping) and encourage couples to desist from resorting to negative coping strategies (like active-avoidant coping) [35].

### 2.1 The myth of “psychogenic sterility”

Evidence-based scientific research [42] has established that the psyche has no direct influence on the causes of infertility (in the sense of “psychogenic sterility”), for example unconscious anxieties about pregnancy/parenthood or a conflictual attitude to the child [40]. Nor should the impact of everyday stress or emotional strain (over-anxiety, depressive states, etc.) on the inception of pregnancy be overestimated [4]. Indirect influence by the psyche may however be an operative factor in behaviourally conditioned sub-fertility. Excessive sport and/or an eating disorder can lead to hypothalamic amenorrhea, which impairs fertility [2]. A behaviourally conditioned fertility disorder is present when

1. despite counselling by a doctor, a couple continues to display behaviour detrimental to fertility (notably over- or underweight, eating habits, seriously competitive sport, overindulgence in nicotine or caffeine, abuse of medicines, etc.),
2. a (heterosexual) couple refrains from sexual intercourse on fertile days or displays a non-organic sexual function disorder, or
3. a couple expressly concedes the necessity of medical therapy in fulfilling their desire for a child but fails to embark on such therapy even after a lengthy period of deliberation, e. g. constantly postponing tube blockage tests or a sperm analysis [42].

The myth of “psychogenic sterility” is also compounded with the commonly held assumption that once a couple has given up their desire for a child, pregnancy will frequently occur spontaneously (e. g. at the beginning of an adoption process). There is no evidence-based substantiation for this belief, the number of these spontaneous pregnancies is well below 10 %. Other popular assumptions have to do with the psychic causes of miscarriage. According to the latest guidelines, everyday stress can be ruled out as a cause of miscarriage [33].

### 2.2 Psychological impact of ART on women and men

In most cases, ART is felt by both partners to be a severe emotional strain. The typical pattern here is the “emotional roller coaster”. Phases of optimistic anticipation and hope give way to disappointment and frustration when menstruation sets in again. This treatment-specific stress is a major reason for prematurely terminating ART treatment, even if the prognosis is favourable [14, 51].

As psychological research is mostly conducted in fertility centres, the repercussions of diagnosis and ART are very difficult to distinguish from one another. There are however indications that even *before* ART couples desiring a child display greater mental strain (anxiety, depressive states) than fertile couples [28].

The unfulfilled desire for a child may frequently have a detrimental effect on the love life of the couple in question [41, 53]. Women more often report loss of libido and impaired sexual satisfaction, while men tend to suffer from erectile dysfunction and ejaculation disorders. Both partners complain especially about a loss of spontaneous physical desire occasioned by scheduled sexual activity designed to fulfil the wish for a child [48]. As a rule, however, these sexual disorders do not normally require treatment and in most cases soon disappear again of their own accord.

### 2.3 Specific aspects of third-party reproduction

Founding a family with gametes or embryos donated by others poses specific psychological aspects of its own, notably in connection with the genetic origins of the child as (at least) one other person is potentially involved in the process. This makes engagement with two topics unavoidable: (a) the question of when the child should be enlightened about its origins, and (b) the question of the identifiability of gamete donors.

The latest scientific research indicates that there is hardly any doubt that a child born as a result of third-party reproduction should be apprised of his/her genetic origins in an appropriate way and at an early stage. These children should also have access to reliable information about their genetic origins and the existence of siblings or half-siblings, if any [29, 42]. The practical implication of this is that for psychosocial reasons couples wanting a child should be strongly advised against availing themselves of *anonymous* gamete donation. Given the increasing use of over-the-counter gene tests, the extent to which the anonymity of gamete donors can be guaranteed in future is at all events anything but clear [1].

### 2.4 Development of pregnancies, children and families after ART

In terms of the subjective experience of pregnancy and birth following ART, apprehension in women is not generally higher than in the case of “normal” pregnancies. However, specific anxieties are more marked, particularly if there have been (repeated) miscarriages in the past and these have been experienced as traumatic [22]. Many women are disappointed by the frequency of caesarean section after ART (including singletons), and after birth this intervention may lead to complications like heightened maternal anxiety and breastfeeding problems.

With regard to singletons born following ART, the number of studies on the situation in families with heterosexual and lesbian parents and on single mothers by choice can be regarded as largely satisfactory [17]. In most cases, children growing up in families of this kind display a development somewhere between unremarkable and unusually good, particularly as most of them are “wanted”. By contrast, families with higher birth multiplicity display not only increased medical risks but also psychosocial risks (higher parent separation rates, retarded development in the children, etc.) [42].

Restrictions have to be made in connection with the situation of older “single mothers by choice”. The demands involved in looking after children requiring above-average care (handicapped children, twins, triplets, etc.) may represent a challenge for them if they have no one to turn to for support (grandparents, etc.). The state of research on psychosocial aspects of family planning via embryo donation, surrogate motherhood and anonymous oocyte donation is inadequate [42].

### 2.5 Long-term effects of involuntary childlessness

There have been a number of studies (including the long-term variety) investigating couples who have remained childless against their will. For couples who have been able to set themselves other goals than parenthood and are not socially isolated, the prognosis is good [42]. Women with a persistent desire for pregnancy 3 to 5 years after unsuccessful treatment may be more likely to experience anxiety and depression than women who find new goals in life or become mothers [13].

In one study, the separation rate 10 to 14 years after infertility treatment was 17 %, which is significantly lower than the separation count for this length of partnership in the population as a whole [45]. More than half the women and over a third of the men in this study referred to social support, communication, an open attitude and active confrontation as positive coping factors in the period when they stopped trying for a child.

## 3 Psychosocial infertility counselling

### 3.1 Subject matter and aims of counselling

The subject matter of psychosocial counselling in cases of involuntary infertility has been described by many researchers [6, 7, 34, 49]. Here are some examples:

**“Give scope to the desire for a child and restrict it at the same time.”** This is the central tenet in psychosocial infertility counselling. There is a life “outside the desire for a child” and it is essential to actively go in search of that life and cultivate it. There should be exploration of the way people in the couple’s **social environment** respond to the desire for a child (“avoid constant white lies”). Normalize and depathologize **“negative” feelings**: intensely “negative” feelings like despair after a negative test or rage at the unfairness of the situation, possibly also feelings of guilt or recriminations against the partner (say, for years of indecision about the desire for a child) are completely normal, understandable and acceptable. **Couple communication**: recognise unhelpful role distributions (such as “depressive woman – helpless man”) and make them more flexible [44]. **Sexuality and reproduction**: As most couples report impairment of their sex lives, counselling should do what it can to alleviate the situation. Before treatment starts, the general **overestimation of the “baby take home” rate** should be addressed. Realistic assessment of success prospects makes it necessary to elaborate **road maps** and **set limits** at an early stage [34]. At the **leave-taking stage**, a workable “plan B” is certainly very helpful [52], as are leave-taking rituals.

Primarily, psychosocial infertility counselling should aim at the reduction of emotional stress independently of the further course of ART (childbirth or not) and seek to emphasize the active part patients can take in dealing creatively with the situation. Other aims are realistic enlightenment on the prospects of success with ART, improvement of couple communication (both between themselves and with others) and the elaboration of alternative perspectives (with or without fulfilment of the desire for a child).

Professional societies insist that the psychosocial aspect should be an integral part of infertility counselling before, during and after ART [13]. It is also the medical doctor’s task to address questions from the psychosocial field. Recent studies indicate that depending on availability, the acceptance of psychosocial counselling is high [32]. Counselling organizations (see www.iico-infertilitycounseling.org for more information) are available in a number of countries: BKiD in Germany, BICA in the UK, ANZICA in Australia and New Zealand (to name a few). Psychosocial interventions (learning relaxation techniques, etc.) can be recommended as a coping aid for patients desiring a child and undergoing ART. Only in very rare cases, however, will they increase the probability of pregnancy. Equally controversial from an evidence-based viewpoint are the effects of other psychosocial interventions [21].

### 3.2 Specific counselling needs in cases of involuntary childlessness

The following constellations urgently necessitate infertility counselling, which in these cases should not be offered on a take-it-or-leave-it basis but explicitly and actively recommended:

Vulnerable individuals/couples (previous psychiatric disorders, abuse of addictive substances, special physical or mental needs and requirements, restricted capacity for “informed consent”, etc.) [42]
Presence of a behaviourally conditioned fertility disorder [25]
Response in the form of individual crises (massive self-esteem problems, depressive response displaying a sustained and pronounced obsession with the desire for a child and neglect of other life-goals, etc.) [46]
Response in the form of a crisis in couple dynamics (sexual problems, prolonged ambivalence or indecision with respect to potential medical treatment, etc.) [44]
Prior to invasive medical interventions (e. g. transition from IUI to IVF/ICSI) and prior to pre-implantation measures or prenatal diagnostic measures [11]
During multiple pregnancy (especially prior to fetocide) [37]
in the case of (repeated) miscarriage or stillbirth [20]
in the presence of genetically (co-)conditioned fertility disorders [5]
in the presence of co-morbidities such as endometriosis [43]
Before, during and after treatment involving gamete or embryo donation or surrogate motherhood [31]
before treatment with reproductive medicine in another country [30]
Donors and surrogate mothers should also be urgently advised to avail themselves of psychosocial counselling before, during and after treatment [29]
in couples from markedly pronatalist cultures [42].

On the topic of (absolute and relative) psychological counter-indications for ART, the present guideline states: “From a scientific point of view, there are (still) no clear psychological contraindications for assisted reproductive treatment. Individual decisions should be taken based on the coupleʼs reproductive autonomy and following interdisciplinary consultation about the childʼs best interests” [42, p. 754].

## 4 Conclusions and outlook

In all probability, involuntary childlessness will increase further all over the world. Medical progress suggests that the problem can be solved via ART. Very often, the media also suggest that this situation can successfully be coped with if the participants are prepared to invest enough time, effort and money in dealing with it. In reality, however, the prospects of assisted reproduction are overrated. Accordingly, a lack of success (including miscarriage) is frequently regarded as personal failure on the part of the individual(s) in question. This can of course exacerbate the usually intense emotional pressure that especially women are exposed to as the major figures involved. From a psychosocial viewpoint, it is essential to actively displace the child as the sole desirable aim of ART from the focus both of the couples affected and the specialists attempting to assist them and to define relief for the distress caused by childlessness as the prime goal of ART [15]. In future, the medical options provided by ART will both be used more widely (cf. the increasing establishment of third-party reproduction all over Europe) and become increasingly sophisticated (in the medium term, the use of, say, CRISPR/Cas to assist childless couples/individuals is unlikely to remain taboo). Accordingly, critical ethical discussion on the subject will be imperative. Equally essential will be the establishment of psycho-educative measures and information resources to help involuntarily childless couples/individuals face up to the fact that despite recourse to ART their desire for a child may well remain unfulfilled [52]. Furthermore, as ART remains still inaccessible in many parts of the world (particularly in sub-Saharan Africa), low-cost IVF activism has to be enhanced [19]. To decrease the stigmatisation of infertility in low- and middle-income countries, infertility destigmatisation interventions need to be implemented across all levels (intra- and interpersonal as well as structural) [16].
